# Supplementary material for: “Virtual Surf Booth”: Assessment of a Novel Tool and Data Collection Process to Measure the Impact of a 6-Week Surf Programme on Mental Wellbeing
Source: Int J Environ Res Public Health. 2022 Dec 13;19(24):16732. doi: 10.3390/ijerph192416732 (PMC9779844; doi:10.3390/ijerph192416732)
Supplement: Supplementary file 1 [file ijerph-19-16732-s001.zip › Supplementary File S1_tool screenshots_revised.pdf]

Supplementary File S1 – Online tool screenshots.

Advice page

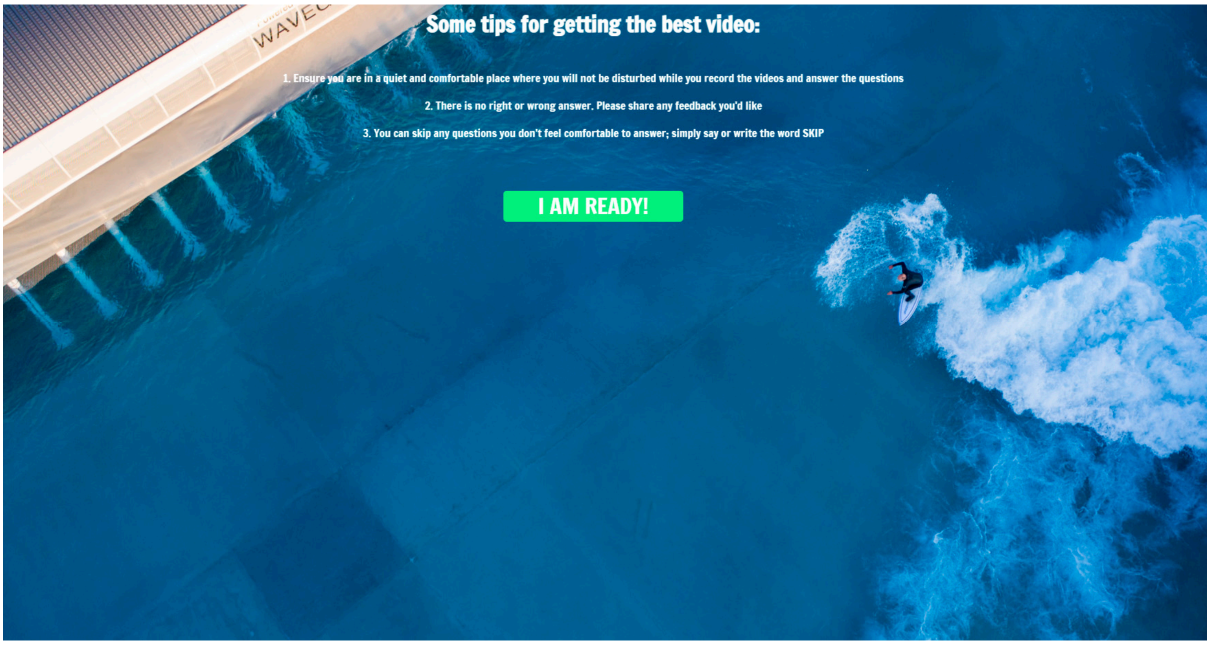

Video Recording S1

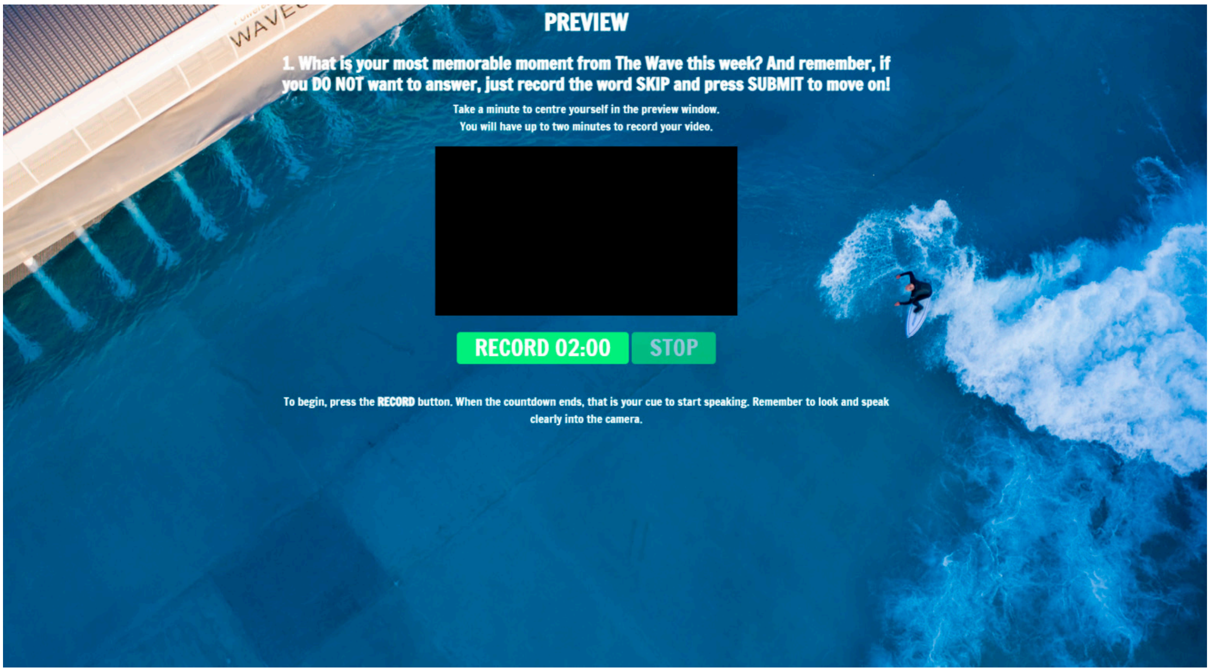

Review Video Recording

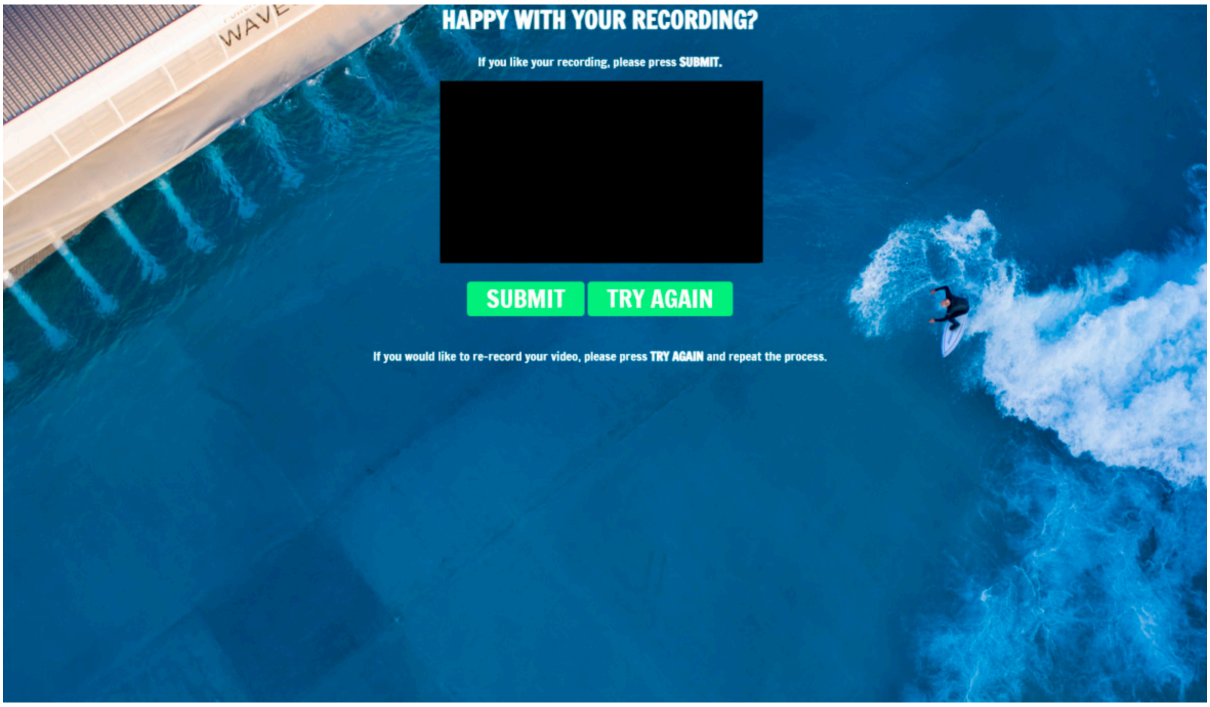

Video Recording S2

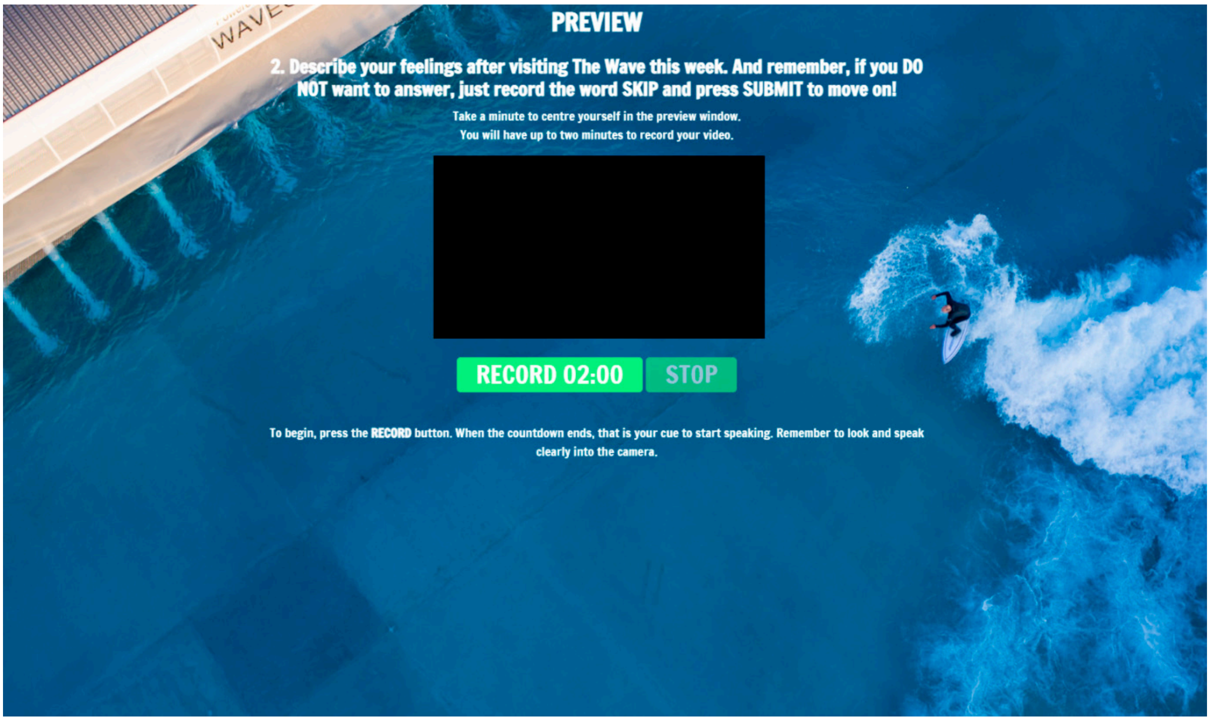

Review Video Recording

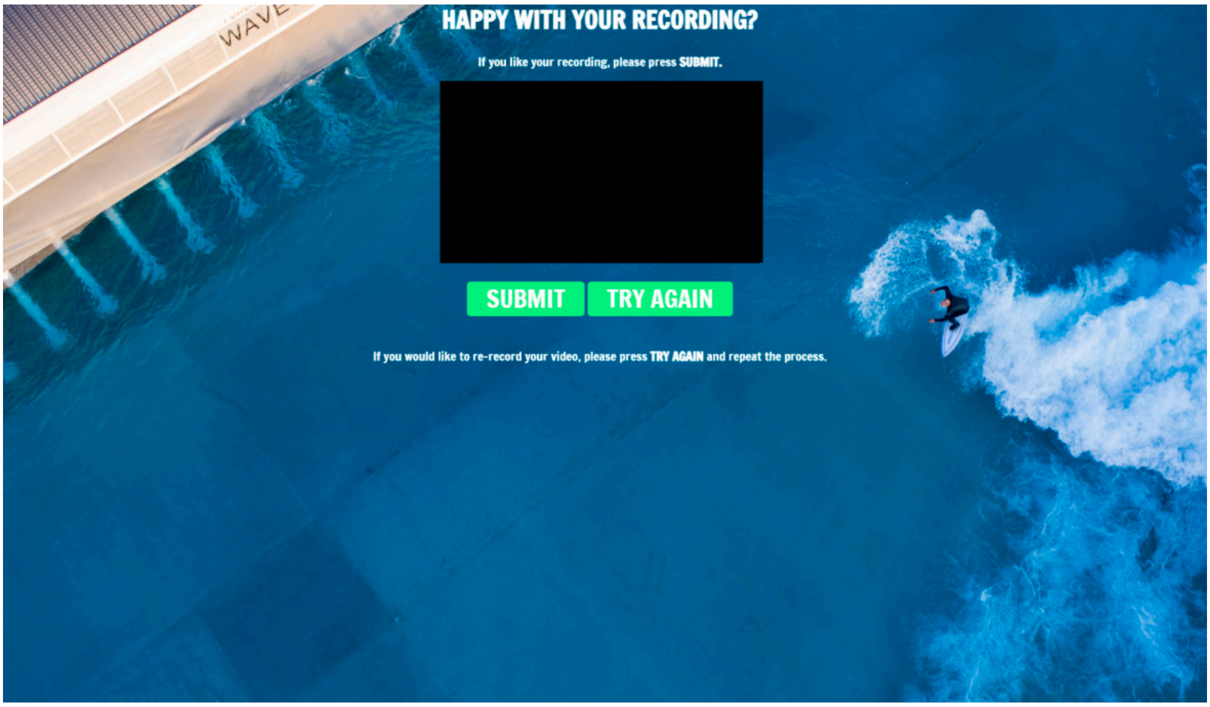

Word Association Question

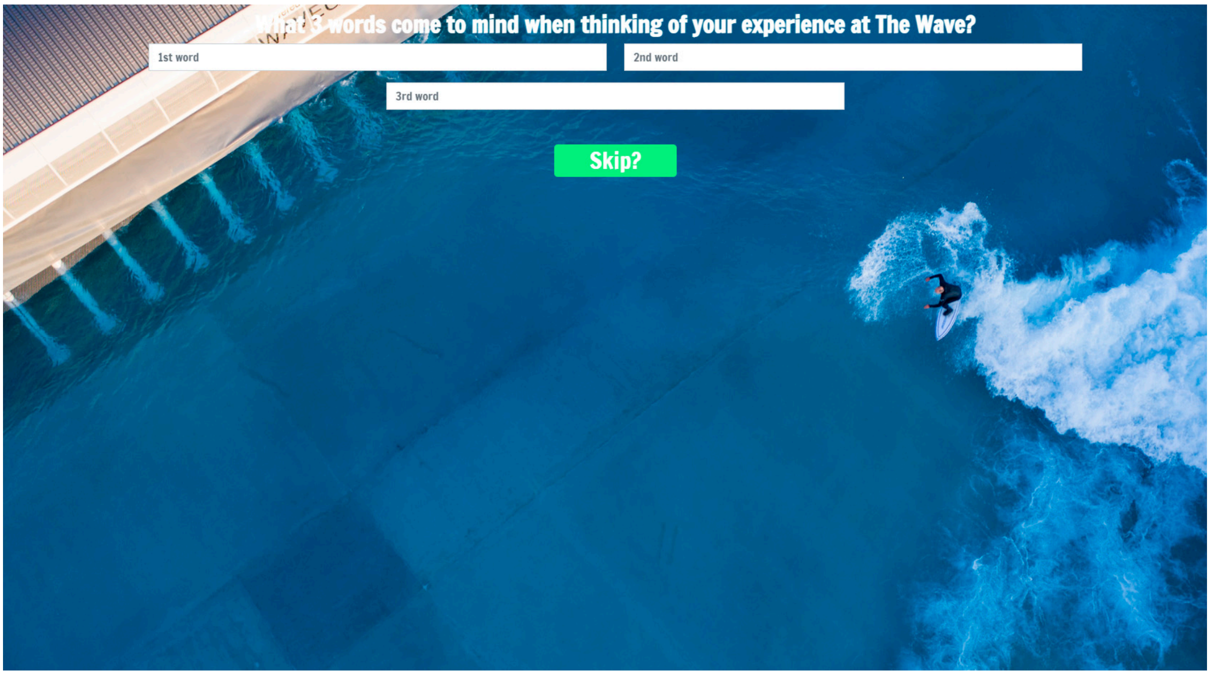

SWEMWBS questions

The Warwick-Edinburgh Wellbeing Scale or [WEMWBS] was developed by the University of Warwick in conjunction with NHS Health Scotland, University of Edinburgh and the University of Leeds. ©University of Warwick, 2006, all rights reserved.

Access to the WEMWBS and SWEMWBS can be requested from this website:
https://warwick.ac.uk/fac/sci/med/research/platform/wemwbs/
