# Supplementary material for: “Virtual Surf Booth”: Assessment of a Novel Tool and Data Collection Process to Measure the Impact of a 6-Week Surf Programme on Mental Wellbeing
Source: Int J Environ Res Public Health. 2022 Dec 13;19(24):16732. doi: 10.3390/ijerph192416732 (PMC9779844; doi:10.3390/ijerph192416732)
Supplement: Supplementary file 1 [file ijerph-19-16732-s001.zip › Supplementary File S2_SWEMWBS outputs.pdf]

Supplementary File S2 – SWEMWBS – descriptive statistics, paired-samples t-test and Wilcoxon signed-rank test analysis.

Descriptive statistics

	Before intervention	After intervention	Change	Positive change?	Statistically significant change?	Wilcoxon signed rank test P value
**Total no. of responses**	11	12				
**% Low wellbeing**	27%	8%				
**% Moderate wellbeing**	64%	58%				
**% High wellbeing**	9%	33%				
**Mean score**	24.3	29	4.60	Yes	Yes	$p<0.05$
**Standard deviation**	6.9	4.0	3.8			
**By age** 25-39	20.0	26.5	6.50	Yes		
40-54	26.7	29.5	2.79	Yes		
**Number of people with a meaningful positive change (%)**				6	60.0	%
**Number of people with a meaningful negative change (%)**				0	0.0	%

Descriptive statistics

	I've been feeling optimistic about the future	I've been feeling useful	I've been feeling relaxed	I've been dealing with problems well	I've been thinking clearly	I've been feeling close to other people	I've been able to make up my mind about things	**Total SWEMWBS score change**	**Positive change?**	**Statistically significant change?**	**Wilcoxon signed rank test P value**
	1	2	3	6	7	9	11				
**Total no. of responses**	12	12	12	12	12	12	12				
**Mean change**	0.09	0.35	1.11	0.66	0.64	0.78	0.61	4.60	Yes	Yes	$p<0.05$

Wilcoxon signed rank test

	n	Sum
**Positive ranks**	8	52
**Total number**	10	
**U_R**	27.5	
**SD**	9.81	
**z**	2.50	
**Significance**	**Yes**	

Paired t test

**Mean**	3.870
**SD**	2.898
**SE (d)**	0.916
**t**	4.223
**df**	9

	Before	After
Mean SWEMWBS score	24.3	28.5

	**Before**	**After**
**% Low wellbeing**	27%	8%
**% Moderate wellbeing**	64%	58%
**% High wellbeing**	9%	33%

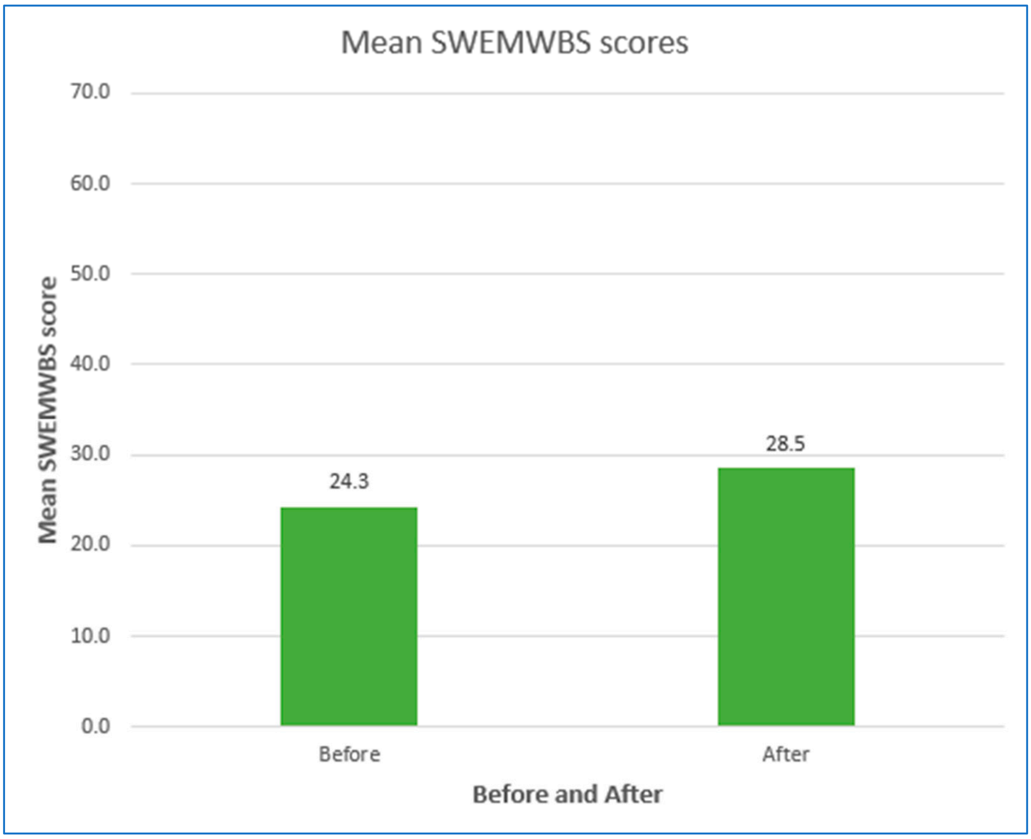
Mean SWEMWBS scores
Mean SWEMWBS score
70.0
60.0
50.0
40.0
30.0
20.0
10.0
0.0
24.3
28.5
Before
After
Before and After

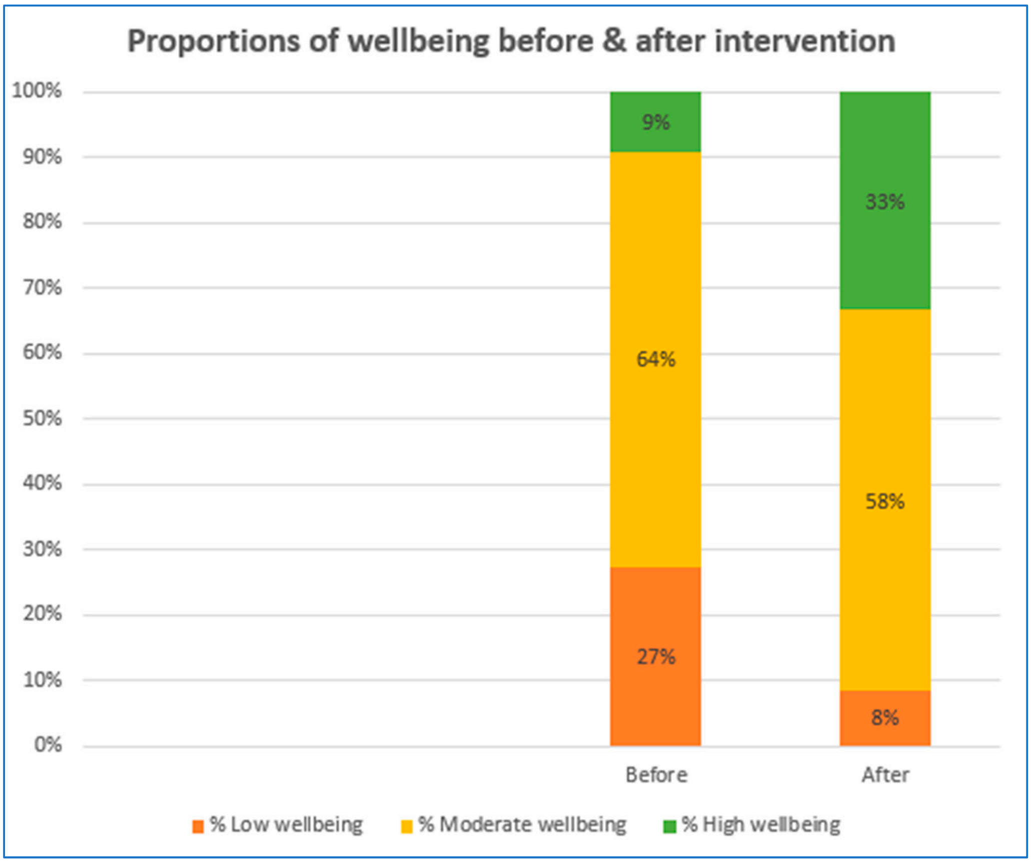

Proportions of wellbeing before & after intervention
100%
90%
80%
70%
60%
50%
40%
30%
20%
10%
0%
9%
64%
27%
33%
58%
8%
Before
After
% Low wellbeing
% Moderate wellbeing
% High wellbeing
