# Supplementary material for: “Virtual Surf Booth”: Assessment of a Novel Tool and Data Collection Process to Measure the Impact of a 6-Week Surf Programme on Mental Wellbeing
Source: Int J Environ Res Public Health. 2022 Dec 13;19(24):16732. doi: 10.3390/ijerph192416732 (PMC9779844; doi:10.3390/ijerph192416732)
Supplement: Supplementary file 1 [file ijerph-19-16732-s001.zip › Supplementary File S3_weather_conditions.pdf]

Supplementary File S3 - weather conditions during the sessions.

**Session**	**Date**	**Weather conditions**
1st	25th of May, 2022	Cloudy, bright, approx. 15°C, evolving towards partly sunny and windy (maximum gust 25 mph)
2nd	8th of June, 2022	Sunny, approx. 17°C
3rd	15th of June 2022	Sunny, approx. 20°C
4th	22nd of June, 2022	Sunny, approx. 23°C
5th	29th of June, 2022	Partly sunny, partly cloudy, drizzling, approx. 17°C, evolving towards rainy
6th	6th of July, 2022	Sunny, 20°C
