# Supplementary material for: “Virtual Surf Booth”: Assessment of a Novel Tool and Data Collection Process to Measure the Impact of a 6-Week Surf Programme on Mental Wellbeing
Source: Int J Environ Res Public Health. 2022 Dec 13;19(24):16732. doi: 10.3390/ijerph192416732 (PMC9779844; doi:10.3390/ijerph192416732)
Supplement: Supplementary file 1 [file ijerph-19-16732-s001.zip › Supplementary File S4_focus groups interview guides.pdf]

**Supplementary File S4 – focus groups interview guides.**

## Research participants

If you did use the online videorecording and survey:

1. Describe how you found using the video recording and survey tool?
    a. Was it easy to use?
    b. Did you need any help to use it?
    c. What did you like/dislike about the tool? The videos?
2. What was your impression on the questions asked?
    a. Easy to understand?
    b. Appropriate to evaluate your mental wellbeing?
    c. How did the questions make you feel?
    d. Would you add/remove any questions, or formulate the questions in another way?
3. What did you think of the frequency of the survey?
4. Can you describe when was the best time and place for you to use it?
    a. After surfing/ In the Wave building/ At home
    b. If you completed the survey at The Wave, did you feel uncomfortable because you were recording in a public space?

If you didn't use the online videorecording and survey:

5. Could you explain why you did not use it?

Whether you used it or not:

6. What could be improved?
7. Would you use this tool if the video recording and survey were conducted in a booth (small room) at The Wave? If not, could you explain why?
    a. If yes, will you share the same information as you did during the study? Or will you share different information? Why?

## The Wave team / participants gatekeepers

1. How did the research project and video recording/survey tool interplay/interact with the surf therapy programme activities?

2. Have the participants shared with you any thoughts or feelings about the research or the tool with you, that you could share with me (without identifying the participant if appropriate)?

3. In your opinion, would the participants use this tool if the video recording and survey were conducted in a booth (small room) at The Wave? Why?
